# Hierarchical Cluster and Region of Interest Analyses Based on Mass Spectrometry Imaging of Human Brain Tumours

**DOI:** 10.1038/s41598-020-62176-8

**Published:** 2020-04-01

**Authors:** Takuya Hiratsuka, Yoshiki Arakawa, Yuka Yajima, Yu Kakimoto, Keisuke Shima, Yuzo Yamazaki, Masahiro Ikegami, Takushi Yamamoto, Hideshi Fujiwake, Koichi Fujimoto, Norishige Yamada, Tatsuaki Tsuruyama

**Affiliations:** 1 Department of Drug and Discovery Medicine, Pathology Division, Kyoto University Graduate School of Medicine, Kyoto, 606-8501 Japan; 2 Department of Neural Surgery, Kyoto University Hospital, Kyoto, 606-8507 Japan; 3 Department of Microbiology, Muroran Institute of Technology, Muroran, Hokkaido 050-8585 Japan; 4 Department of Forensic Medicine, Graduate School of Medicine, Tokai University School of Medicine, Isehara-Shimokasuya 143, Kanagawa, 259-1193 Japan; 5 Kyoto Applications Development Center, Analytical & Measuring Instruments Division, Shimadzu Corporation, 1 Nishino-kyo-Kuwabara-cho, Kyoto, 604-8511 Japan; 6 Research Center, Shimadzu General Services, Inc., 1 Nishino-kyo-Kuwabara-cho, Kyoto, 604-8511 Japan; 7 Clinical bioresource centre, Kyoto University Hospital, Kyoto, 606-8507 Japan

**Keywords:** Proteomics, Tissue engineering, Neurological disorders, Biological techniques, Biotechnology, Neurology, Oncology, Cancer, Analytical chemistry, Bioanalytical chemistry, Imaging studies, Mass spectrometry, Medical and clinical diagnostics

## Abstract

Imaging mass spectrometry (IMS) has been rarely used to examine specimens of human brain tumours. In the current study, high quality brain tumour samples were selected by tissue observation. Further, IMS analysis was combined with a new hierarchical cluster analysis (IMS-HCA) and region of interest analysis (IMS-ROI). IMS-HCA was successful in creating groups consisting of similar signal distribution images of glial fibrillary acidic protein (GFAP) and related multiple proteins in primary brain tumours. This clustering data suggested the relation of GFAP and these identified proteins in the brain tumorigenesis. Also, high levels of histone proteins, haemoglobin subunit α, tubulins, and GFAP were identified in a metastatic brain tumour using IMS-ROI. Our results show that IMS-HCA and IMS-ROI are promising techniques for identifying biomarkers using brain tumour samples.

## Introduction

Recently, matrix-assisted laser desorption/ionization-time of flight (MALDI-TOF) mass spectrometry has been used to identify diagnostic markers. MALDI-TOF imaging mass spectrometry (MALDI-IMS) now helps to identify phospholipids^1,2^ delivered drugs^3^, and peptides in various tissues^4–7^. Breast tumour tissue was used in a recently reported study of MALDI-IMS^8^. Reports have also been presented regarding IMS being used in the analysis of gastrointestinal, larynx, and ovarian tumours^9,10^, as well as other diseases^11^. In our previous study, we also succeeded in identifying proteins related to important myocardial functions such as ATP synthase in acute myocardial infarction^12–14^.

In the recent five years, clinical formaldehyde-fixed paraffin-embedded (FFPE) tissue has been made available for IMS studies^2,12,15–17^. Formaldehyde reacts with amino acid residues, such as arginine-containing amino groups, by methylene-bridging. The bridge makes it challenging to ionize peptides, and study is therefore difficult when using FFPE for IMS. For this reason, alcohol-based non-crosslinking tissue fixative could be an alternative fixative for multiomics tissue analysis, but its usefulness has not been fully verified^18^. Some studies have reported that the use of surfactants improved MS sensitivity using considerable limits on subjects and sample amounts for a stable protocol^14^. Angel *et al*. described the availability of matrix metalloproteinase enzymes to derive a better signal^19^.

In the current study, glioblastoma was selected as the disease of interest for IMS study using FFPE. Glioblastoma is one of the most aggressive brain tumours^20^. Treatment of glioblastoma includes a multidisciplinary approach that provides for maximal surgical resection, radiation therapy^21^, and chemotherapy^22,23^. The last two therapies primarily target metastatic carcinoma and malignant lymphoma^24^. For better treatment, it is crucial to differentiate glioblastoma from metastatic carcinoma and malignant lymphoma. Moreover, for earlier diagnosis, proteomic approaches^25^ to substances in the blood of glioblastoma patients have become prevalent^26^. IMS analysis has been applied to mouse models of brain diseases^27,28^ as well as glioblastoma for pharmacokinetics, metabolomics, and lipid analysis^9,29–31^. However, reports of proteomic markers by IMS of glioblastoma remain rare. Here the analytical algorithm method has been further developed, using IMS combined with hierarchical cluster analysis (IMS-HCA) for recognition of the distribution pattern of ions ionized and MS combined with region of interest (IMS-ROI) analysis for quantification of the signals, to identify the tumour and determine the invasion range.

## Results

### Histopathology and IMS analyses

**Figure 1:**
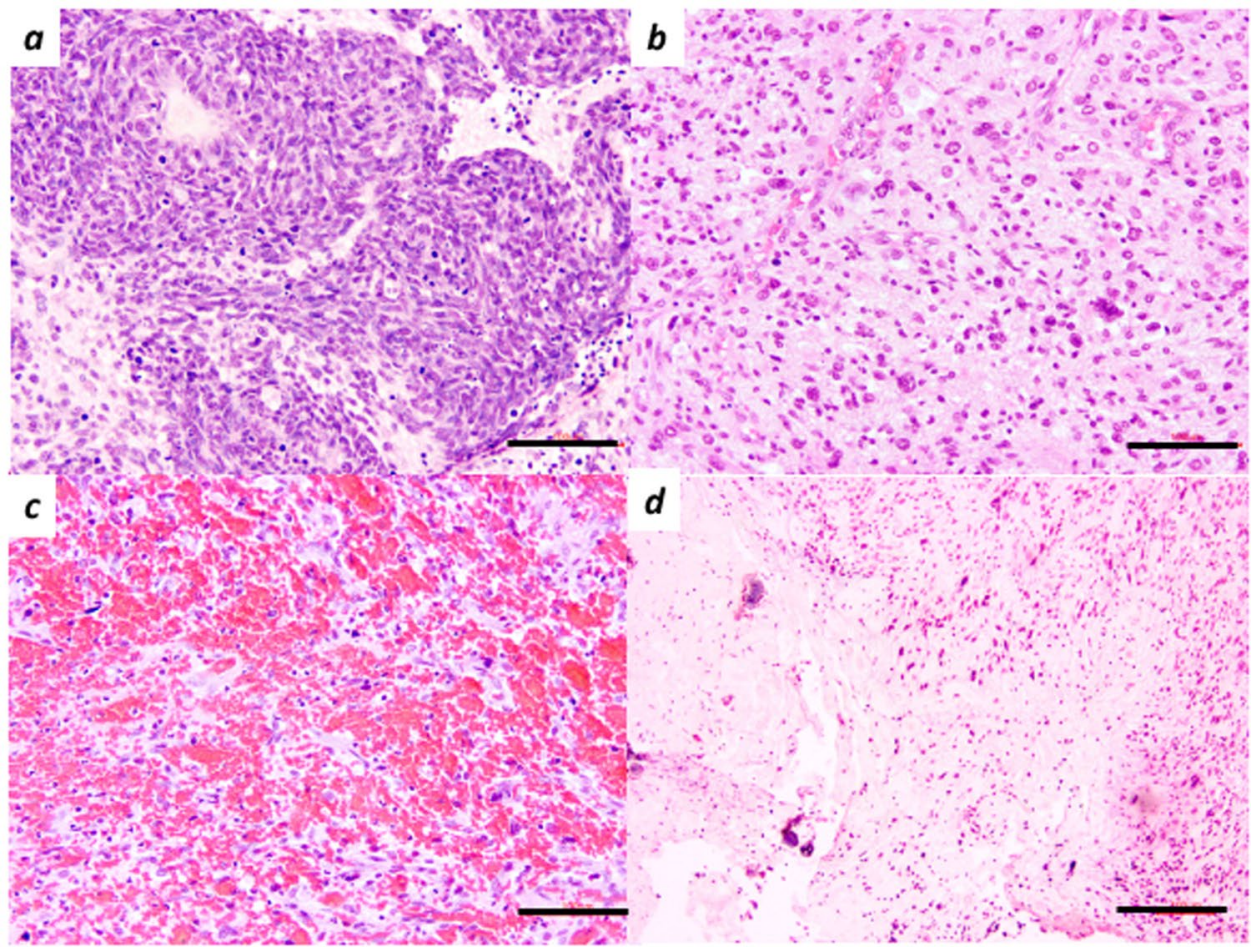
Histological images of metastatic lung small cell carcinoma (**a**) and glioblastoma (**b**–**d**) in haematoxylin and eosin staining. (**a**) (sample 1) Metastatic small cell lung carcinoma has a dense sheet of small cells with scanty cytoplasm, and finely granular nuclei. (Original magnification: X200). Scale bars, 100 µm in (**a**–**d**). (**b**) (sample 4) Cellular lesion of glioblastoma shows high cellularity, prominent cytologic atypia and pleomorphism. (Original magnification: X200) (**c**) (sample 4) Haemorrhage among tumour cells. (Original magnification: X200) (**d**) (sample 4) Necrosis is surrounded by the pseudo-palisading arrangement of nuclei of tumour cells. (Original magnification: X100).

3 glioblastoma and 1 brain metastasis of the small cell lung carcinoma (SCLC) samples were chosen for our study because of the high quality. The morphologic feature of SCLC (Sample 1) was a dense sheet of small cells with ill-defined contour, finely granular nuclei, and inconspicuous nucleoli^4^ (Fig. 1a). Glioblastoma is characterized by high cellularity, pleomorphism, a frequent mitotic number, the presence of vascular proliferation, and the existence of necrosis^3^. Three specimens of glioblastoma show some of these characteristics (Fig. 1b, c, d).

### MALDI-IMS analyses

**Figure 2:**
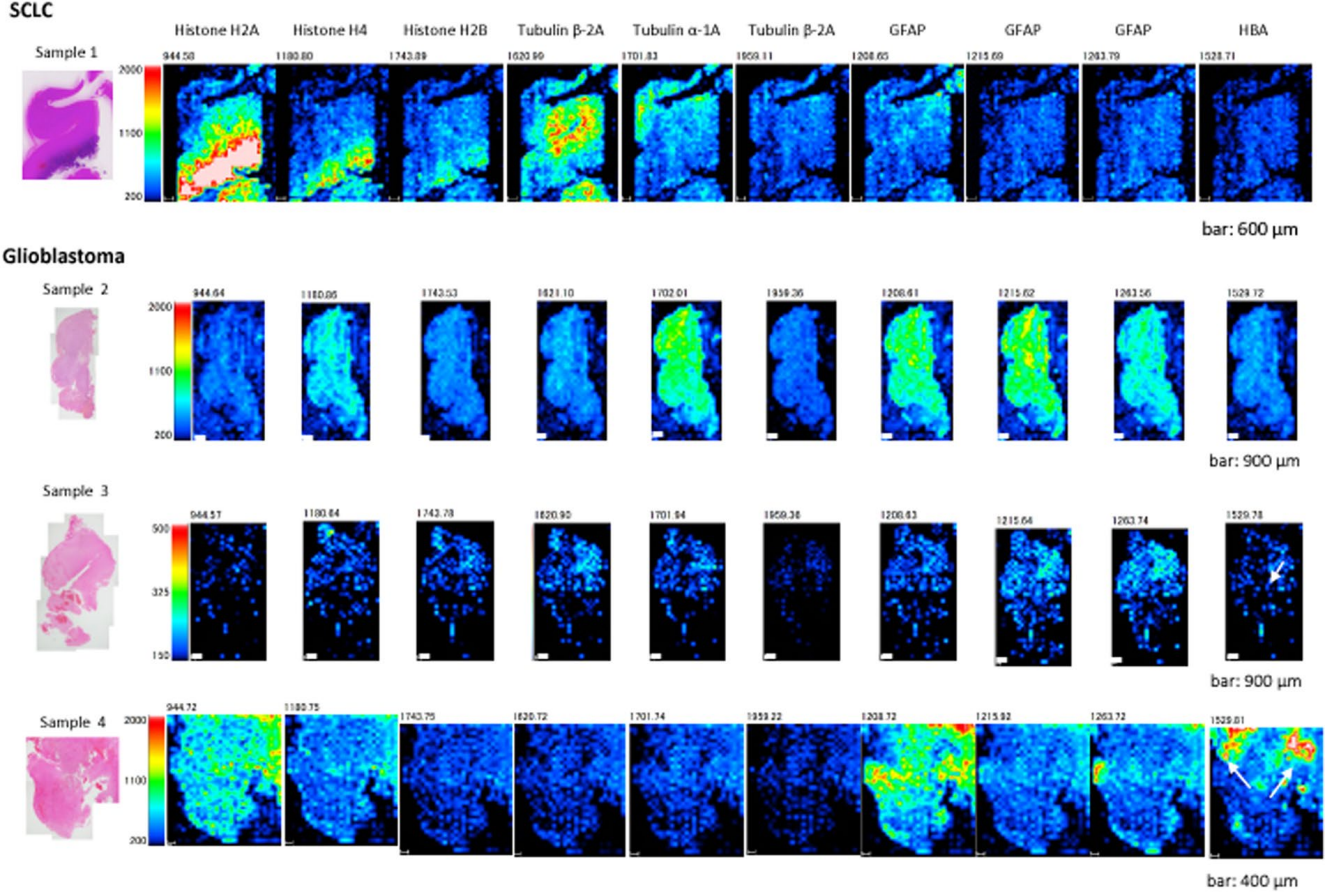
MALDI-IMS analyses of samples. Each identified protein is shown at the top. Each *m/z* value is noted above the images of samples 1–4. Scale bars: 600 μm (sample 1), 900 μm (samples 2 and 3), or 400 μm (sample 4). Each identified protein is shown at the top. The small white arrow indicates the haemorrhagic region in sample 3 and sample 4. SCLC, small cell lung cancer.

**Figure 3:**
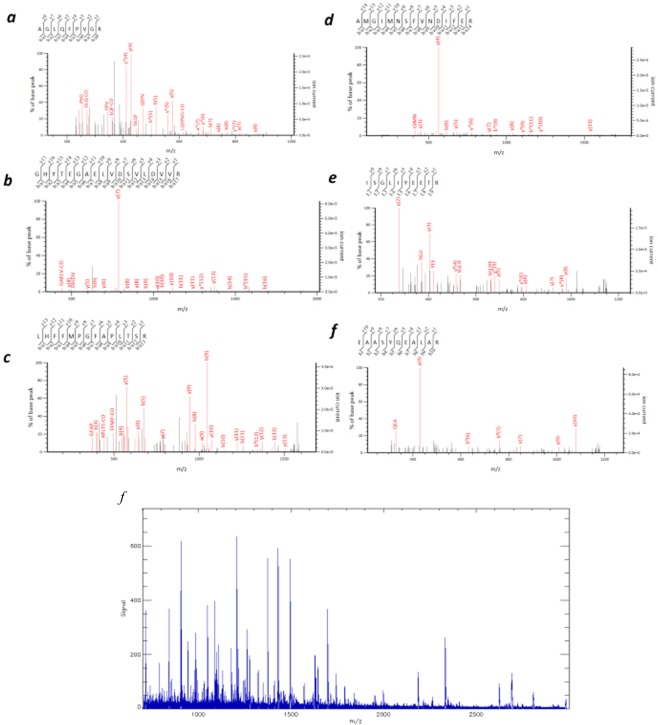
MS profiling of SCLC and glioblastoma. (**a**) Tandem mass spectrum of the precursor ion in SCLC (sample 1) at *m/z* 944.55. A database search identified the ion at *m/z* 944.55 as a fragment of Histone H2A type 1-A, *P* = 0.024, (**b**) Tubulin α1-A, *m/z* 1701.92, *P* = 0.0094, (**c**) Tubulin β2-A, *m/z* 1620.94, *P* = 1.4 × 10^−5^, (**d**) Histone H2B type 1-B, *m/z* 1743.75, *P* = 0.051, (**e**) Histone H4, *m/z* 1180.66, *P* = 5.1 × 10^−5^, (**f**) Glial Fibrillary Acidic Protein (GFAP), *m/z* 1208.6, *P* = 0.029. (**g**) MS profiling of glioblastoma (sample 2).

Samples of glioblastoma and metastatic SCLC were analysed by MALDI-IMS. Glioblastoma and SCLC samples were analysed separately via LC/MS or tandem MS/MS across the *m/z* range of 700–3000. We obtained MALDI-IMS signals that corresponded to a total of >500 peptides. Of these signals, approximately 50 spectra had sufficient intensity to obtain IMS data in each sample. MALDI-IMS and MS/MS data for SCLC brain tissue are shown in Figs. 2 and 3a–f, respectively. An example of the total spectrum of the glioblastoma (sample 2) is shown in Fig. 3g.

In Sample 1, proteins with *m/z* values of 944.58 (Histone H2A), 1180.80 (Histone H4), 1743.89 (Histone H2B), 1701.83 (Tubulin α-1A), 1959.11 (Tubulin β-2A), 1208.65 (GFAP: glial fibrillary acidic protein) were identified via tandem MS/MS (Fig. 3a–f). As shown in the first row of Fig. 2 (SCLC), the signals of Histone H2A, Histone H4, and Histone H2B were found in accordance with the histopathological distribution of SCLC (Fig. 2). We created the MALDI-IMS in the range of a tolerance level of ±0.5 Da for each *m/z* gained in sample 1.

**Table 1:** List of peptide peaks identified by MS/MS analysis in the FFPE human brain tissue.

Observed *m/z*	Sequence	Protein name	Expect (P < 0.05)
944.55	R.AGLQFPVGR.I	Histone H2A type 1-A (H2A1A_HUMAN)	0.024
1180.66	R.ISGLIYEETR.G	Histone H4 (H4_HUMAN)	5.10E-05
1743.75	K.AMGIMNSFVNDIFER.I	Histone H2B type 1-B (H2B1B_HUMAN)	0.051
1620.94	R.LHFFMPGFAPLTSR.G	Tubulin beta-2A chain (TBB2A_HUMAN)	1.40E-05
1620.78	R.LHFFMPGFAPLTSR.G	Tubulin beta-2A chain (TBB2A_HUMAN)	1.10E-05
1701.92	R.AVFVDLEPTVIDEVR.T	Tubulin alpha-1A chain (TBA1A_HUMAN)	0.00059
1959.22	K.GHYTEGAELVDSVLDVVR.K	Tubulin beta-2A chain (TBB2A_HUMAN)	0.0094
1208.60	R.EAASYQEALAR.L	Glial fibrillary acidic protein (GFAP_HUMAN)	0.029
1208.64	R.EAASYQEALAR.L	Glial fibrillary acidic protein (GFAP_HUMAN)	0.036
1215.68	R.DNLAQDLATVR.Q	Glial fibrillary acidic protein (GFAP_HUMAN)	0.025
1263.73	R.LEAENNLAAYR.Q	Glial fibrillary acidic protein (GFAP_HUMAN)	0.00037
1529.74	K.VGAHAGEYGAEALER.M	Hemoglobin subunit alpha (HBA_HUMAN)	7.10E-05

The signal of the glial fibrillary acidic protein (GFAP) was identified in samples 2–4 from the total spectrum by inputting the above *m/z* values. The IMS of haemoglobin subunit α(HBA) was obtained in the haemorrhagic region of sample 4 shown in Fig. 2 (indicated by arrows). Furthermore, IMS of Histone H2A, Histone H4, Histone H2B, Tubulin α-1A, and GFAP were obtained in the glioblastoma tissues (sample 2–4) (Fig. 2).

### IMS combined with HCA for the selected proteins

**Figure 4:**
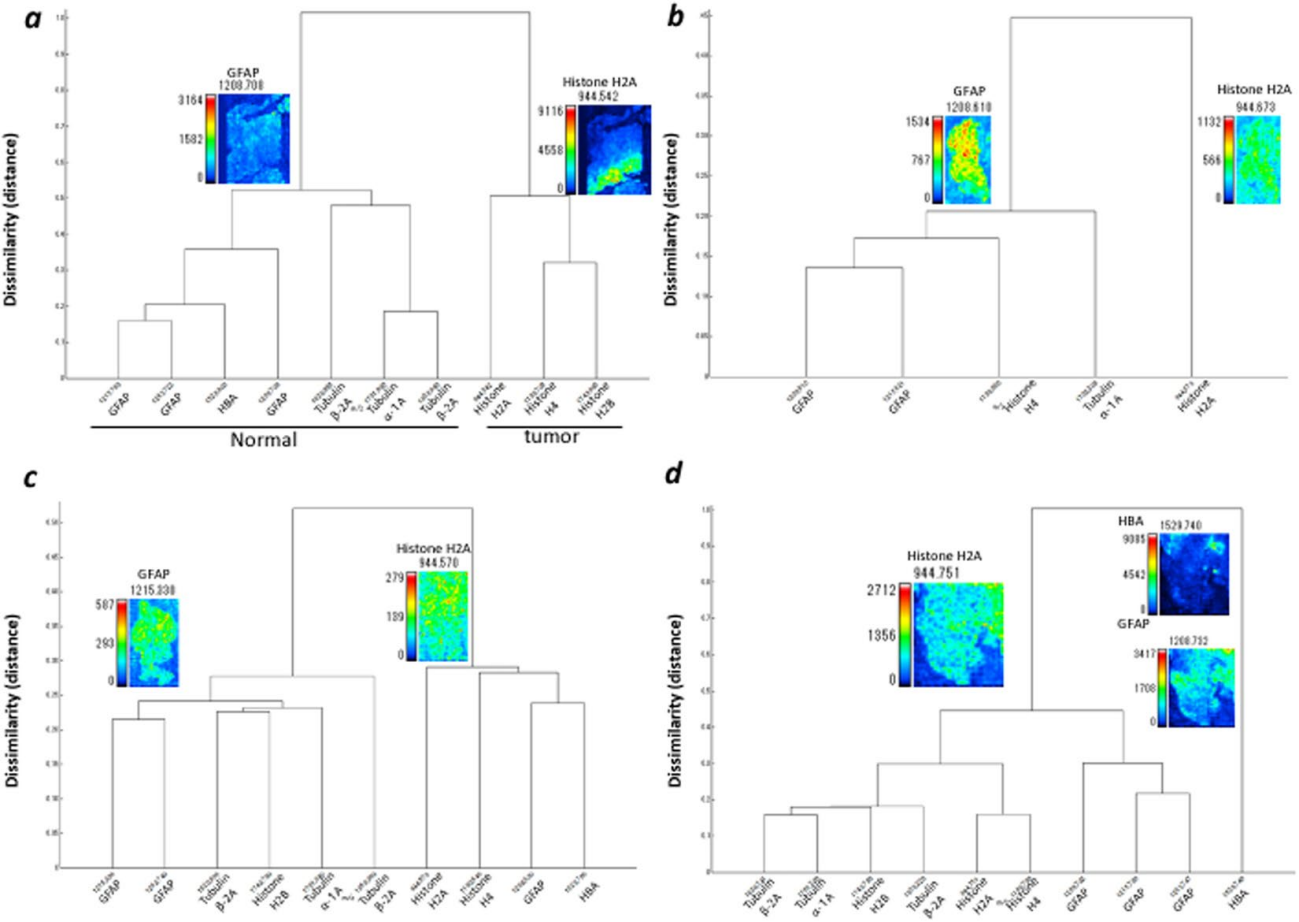
Cluster analysis of the two-dimensional distribution of the selected peptides. The data from two-dimensional distributions were used to calculate a cluster dendrogram. The vertical axis represents dissimilarity, while the *m/z* values are represented on the horizontal axis. HCA analysis, with Ward method (**a**) sample 1, (**b**) sample 2, (**c**) sample 3, and (**d**) sample 4.

First, MALDI-IMS combined with HCA (IMS-HCA) with Ward method was performed using metastatic tumour sample 1. IMS-HCA showed a cluster including Histone H2A, H4, and Histone H2B (Tumour, Fig. 4a). In contrast, other proteins from normal region belonged to different clusters (Normal, Fig. 4a), indicating that IMS-HCA method was available for distinction of the tumour region from the normal region. In sample 2, IMS-HCA with Ward method showed corresponding clusters of GFAP, Histone H4, and Tubulin α-1A (Fig. 4b). In sample 3, consistent with the IMS data in Fig. 2, the signals of GFAP, Tubulin α-1A, Histone H2B and Tubulin β-2A formed a close cluster in IMS-HCA with Ward method (Fig. 4c). Also, the HBA signal in the cerebral blood vessels away from the glioblastoma region formed a cluster separate from the cluster of GFAP signals. The HBA clustering result revealed that glioblastoma and blood vessel placement are irrelevant in this specimen (Fig. 4d). As described above, the correlation between two-dimensional distribution of signal intensity in IMS data and histopathology could be well verified by IMS-HCA. The HCA of samples 1, 2, 3, and 4 with group average method showed the same clustering results regarding the relationship among the clusters to which the proteins belonged, except the dissimilarity (distance) as demonstrated in Supplementary Fig. 1a–d.

### IMS-HCA for the total proteins

**Figure 5:**
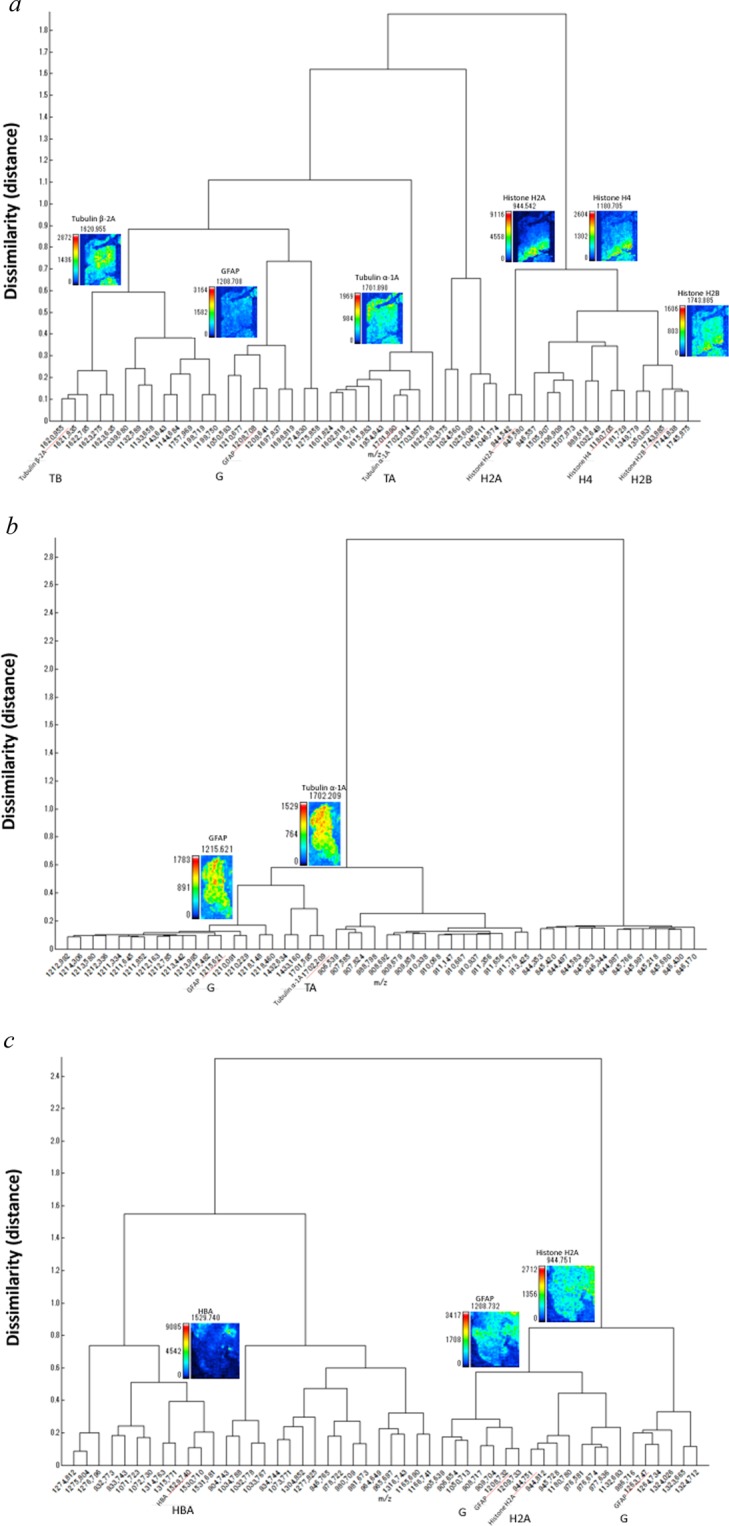
Cluster analysis of the two-dimensional distribution of the whole spectrum. The data from two-dimensional distributions were used to calculate a cluster dendrogram. The vertical axis represents dissimilarity, while the *m/z* values are represented on the horizontal axis. HCA analysis with Ward method: (**a**) sample 1, (**b**) sample 2, (**c**) sample 4. HB, HBA; TB, Tubulin β-2A; G, GAFP; TA, Tubulin α-1A; H2A, Histone H2A; H4, Histone H4.

First, IMS-HCA was performed using metastatic SCLC tumour sample 1 with Ward method (Fig. 5a). IMS-HCA showed a corresponding cluster of Histone H2A, Histone H4, and H2B, whereas GFAP and Tubulin β-2A belonged to different clusters. This result is compatible with that of IMS-HCA for the selected proteins in Fig. 4a. Next, in sample 2, IMS-HCA with Ward method showed that the GFAP and Tubulin α-1A belonged to clusters, which was compatible with the above IMS-HCA for the selected proteins in Fig. 4b (Fig. 5b). In sample 3, IMS-HCA with Ward and other methods could not obtain clustering because of the lower intensity of the signal. In sample 4, IMS-HCA formed a close cluster of Histone H2A and GFAP, and distant cluster of HBA and GFAP (Fig. 5c). The HCA of samples 1, 2, and 4, with group average method, showed the same clustering results, regarding the relationship between the proteins except the dissimilarity (distance) as shown in Supplementary Fig. 2a–c.

### IMS-ROI quantification of peptides in tumour region

**Figure 6:**
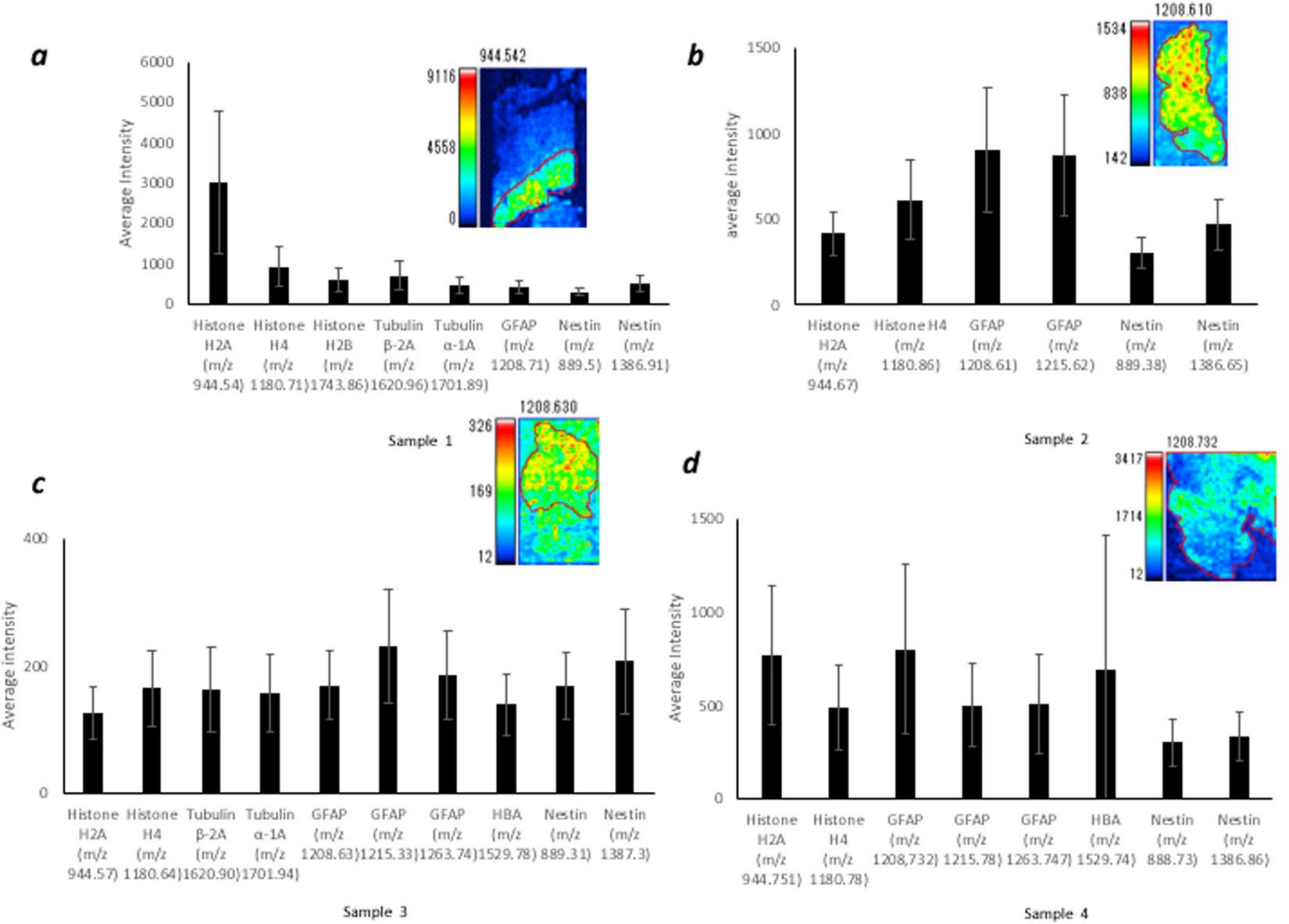
Quantification of peptides in the tumour region. On the vertical axis, the average intensity in tumour region of (**a**) sample 1, (**b**) sample 2, (**c**) sample 3, and (**d**) sample 4 is shown, and on the horizontal axis, the *m/z* values of individual protein-derived peptides are shown. (**a**) IMS of Histone H2A and (**b**–**d**) IMS of GFAP are shown.

For MALDI-IMS-ROI (IMS-ROI) analysis, the tumour region was independently margined by three pathologists, and the ROI was nearly shared. We measured the intensity of the signals of Histone H2A, Histone H4, Histone H2B, Tubulin β-2A, Tubulin α-1A, GFAP, and HBA using Imaging MS Solution (sample 1: N = 369, sample 2: N = 514, sample 3: N = 532, sample 4: N = 925). These statistics are shown in Table 2. The average intensities of Histone H2A and Histone H4 were significantly higher than other peptides in the metastatic tumour region in sample 1. Specifically, the average intensity of GFAP was lowest in all peptides except Nestin (Fig. 6a, Supplementary Table 1).We found the average intensity of GFAP was significantly higher than those of Histone H2 and Histone H4 (Fig. 6b, Supplementary Table 1) in sample 2. The average intensity of Histone H2A, Histone H4, Histone H2B, Tubulin β-2A, Tubulin α-1A, and GFAP in sample 3 was high in the ROI (Fig. 6c, Supplementary Table 1). In sample 4, we found that the average intensities of Histone H2A and GFAP (*m/z* = 1208) were significantly higher. Additionally, the average intensity of HBA was high in the tumour region, indicating that the aforementioned haemorrhage in the tumour region (Fig. 6d, Supplementary Table 1). GFAP was high in the tumour regions of all glioblastoma samples.

**Table 2:** Statistical analysis using IMS-ROI. Tumour (T) and Normal (N) represent intensity.

Sample 1 *m/z*	Mean (T)	SD (T)	Mean (N)	SD (N)	*p* value	*d* value	Effect
Histone H2A(*m/z* 944.542)	3015.8	1777.8	794	512.4	5.20E-93	2	significant
Histone H4 (*m/z* 1180.705)	917.3	500	408.8	174.8	4.95E-73	1.6	significant
Histone H2B (*m/z*1743.885)	589.9	287.1	414.5	176.3	3.12E-25	0.8	significant
Tubulin β-2A (*m/z* 1620.955)	693.4	355.5	922.7	538.4	1.87E-11	0.5	significant
Tubulin α-1A (*m/z* 1701.890)	452.6	188.1	659.6	310.6	1.24E-32	0.8	medium
GFAP (*m/z* 1208.708)	402.1	150.5	503.7	234.3	1.09E-15	0.5	medium
**Sample 3** ***m/z***	**Mean** (**T**)	**SD** (**T**)	**Mean** (**N**)	**SD** (**N**)	***p*** **value**	***d*** **value**	**Effect**
Histone H2A (*m/z* 945.728)	125.9	40.8	96.3	36.6	2.64E-39	0.26	small
Histone H4 (*m/z* 1181.735)	164.9	59.9	123.4	48.4	1.44E-58	0.5	medium
Tubulin β-2A (*m/z* 1621.380)	162.9	65.2	110.6	48.8	2.87E-95	0.31	small
Tubulin α-1A (*m/z* 1703.420)	157	60	111.3	47.2	6.14E-92	0.3	small
GFAP (*m/z* 1208.732)	169.4	54.1	128.2	50.5	3.38E-65	0.24	small

### Comparison of peptides in tumour and normal region by IMS-ROI analysis

We calculated the average intensity of peptides by IMS-ROI analysis using Imaging MS Solution. In samples 1 and 3, each sample contained both tumour and normal tissue. The tumour and normal regions were selected as the ROI. We measured the intensity of the signals of Histone H2A, Histone H4, Histone H2B, Tubulin β-2A, Tubulin α-1A and GFAP (*m/z* 1208) in each ROI (sample 1 tumour: N = 369, normal: N = 730; sample 3 tumour: N = 532, normal: N = 231).

**Figure 7:**
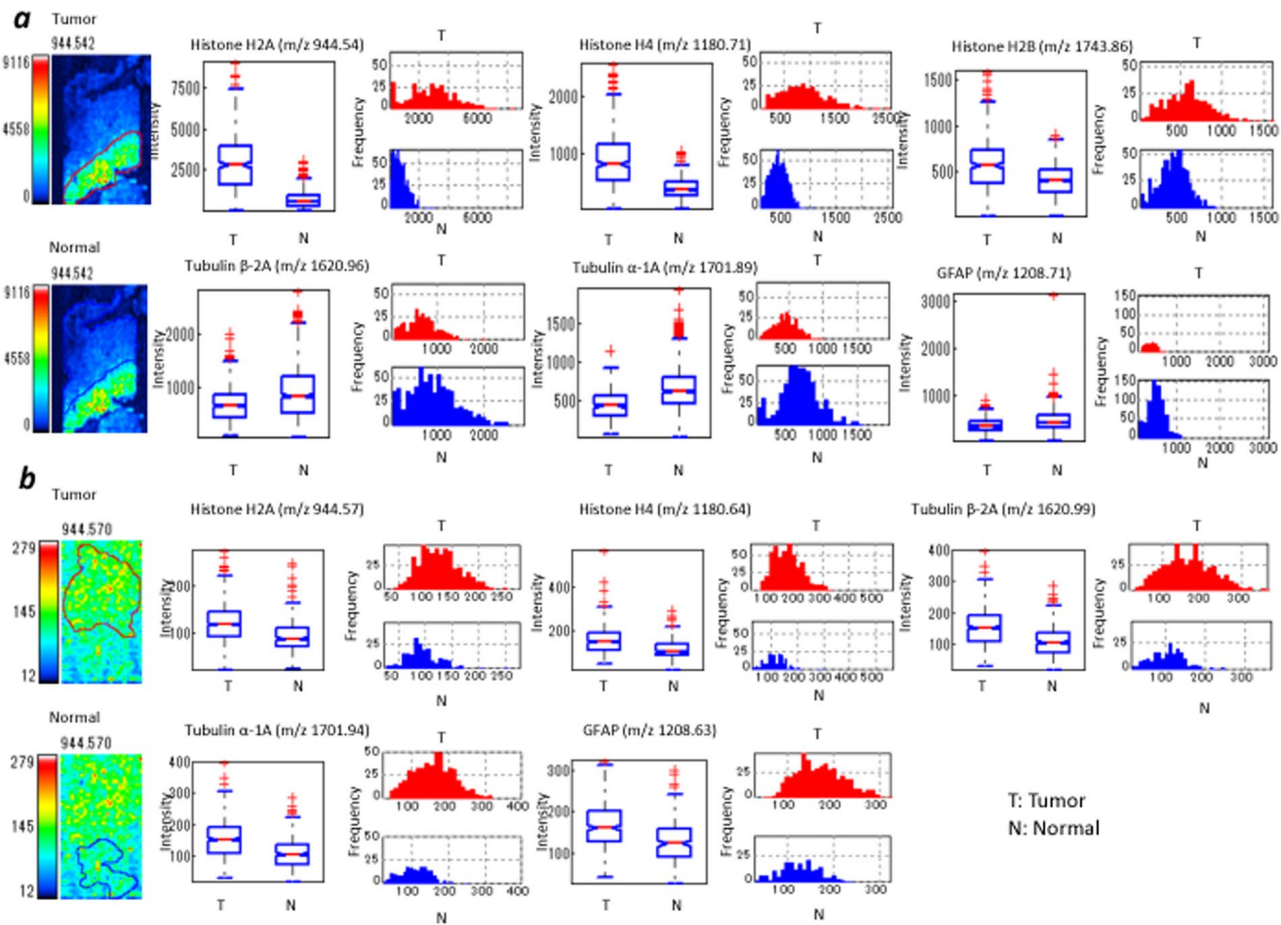
Quantification of peptides in tumour and normal regions by IMS-ROI analysis. (**a**) sample 1, (**b**) sample 3. The box plot shows the distribution of intensity of all pixels in a *m/z* image in each tumour and normal region. Box plot explanation: upper horizontal line of box, 75th percentile; lower horizontal line of box, 25th percentile; horizontal bar within box, median; upper horizontal bar outside box, 90th percentile; lower horizontal bar outside box, 10th percentile. The histogram shows the frequency of intensity of all pixels of the *m/z* image each tumour and normal region. The histogram vertical axes represent the frequency of pixels. The horizontal axes represent intensity. The two images on the left side show each ROIs. The red lines express the border of the ROI for tumour region. The blue lines express the ROI of the normal region. The image at the left coloum represents IMS of Histone H2A.

According to Cohen’s *d*-value-based IMS-ROI analysis, in sample 1, we observed that the average intensities of Histone H2A, Histone H4, Histone H2B, and were higher in the tumour region than those in the normal region (*P*- and *d-*values; significant and medium, respectively, in Table 2). The average intensities of Tubulin β-2A, Tubulin α-1A and GFAP (*m/z* 1208) were lower in the metastatic tumour region than in the normal region (small) (Fig. 7a and Table 1).

Similarly, in sample 3, we found the average intensities of not only Histone H2A, Histone H4, Histone H2B, Tubulin β-2A, and Tubulin α-1A, but also GFAP (*m/z* 1208) were higher in the glioblatoma region than the normal region (all proteins for *P*-values, Histone H4 for *d*-value) (Fig. 7b and Table 1). The standard deviation of intensity data in Fig. 7 was significantly variable than that of the normal region as shown in the boxplot, suggesting the pleomorphism of glioblastoma region.

## Discussion

IMS research has recently made significant progress in determining the distribution of small, specific molecules in tissues, namely in the fields of lipid identification and brain tumor cell clusters in brain tissue. On the same token, it has now found use in peptide identification. Our studies show that the IMS method has been extended to increase the usability of FFPE tissue significantly.

Our application of imaging MS Solution has facilitated the evaluation of the signal intensity distribution patterns. The similarity between clusters was assessed via the geometric distance between the pixels. There are several methods for clustering. Herein, we consider that the Ward methods, and the group average method are reasonable because these methods analyze all pixels’ intensity. In contrast, only a part of the pixels was subjected to the analysis in other clustering methods. The group average method is a method in which the average of the inter-sample distances of all combinations between clusters is set as the inter-cluster distance. The Ward method is as follows. First, assume a new cluster that combines two clusters. Next, let *L* be the sum of squares of the distance between the centre of gravity of the new coupled-cluster and each sample. Next, let *L* (P) and *L* (Q) be the sum of the squares of the distance between the centre of gravity of the two original clusters and the sample inside. Finally, clusters that minimize *Δ* = *L* − *L* (P) − *L* (Q) are combined to obtain a new cluster. Forming a new connected cluster repeats minimizing errors because the information is reduced compared to the original cluster distribution. The Ward method reflects the actual state of the cluster more quickly than the group average method and it is often used as a default. The Ward method is employed because a more general result can be obtained compared to the group average method. However, in the current study, the Group average method and Ward methods showed similar clustering (Figs. 4, 5, Supplementary Figs. 1, 2). We identified that 50 of the 500 peptides’ spectra in the HCA show high signal intensity. However, many did not meet our pre-set statistical accuracy requirements in the current study. Therefore, only the proteins shown in the cluster tree now satisfy the statistical accuracy. The reason for the lack of statistical accurtuacy was that the short peptide sequence could not completely rule out the possibility of other proteins according to Mascot search engine (see Methods).

Furthermore, we adopted IMS-HCA to evaluate the two-dimensional distribution information more accurately. Each pixel *m/z* data set provides similarities between peptide distribution patterns. Of particular note is that Histone H2A belongs to the tumour cluster in both metastatic SCLC. Previous studies have shown that Histone H2A is highly detectable by mass spectrometry of colorectal cancer tissue^2^. In both IMS-HCA for the selected protein (peptide) in Fig. 4 and IMS-HCA for whole peptides in Fig. 5, cluster classification that closely matched the two-dimensional distribution pattern of IMS was confirmed. The ability for pattern similarity to be expressed quantitatively by the distance between clusters indicates a correlation between peptides that belong to closer clusters. GFAP is one of the well-known diagnostic markers for glioblastoma, but the proteins forming clusters close to those of GFAP may relate to the pathogenesis of glioblastoma. Here, Tubulin α-1A and Histone H2A belonged to a relatively close cluster of GFAP (Fig. 5b, Tubulin α-1A; Fig. 5c, Histone H2A; Supplementary Fig. 1b, Tubulin α-1A; Supplementary Fig. 1c, Histone H2A). In this way, based on this IMS-HCA data, the distribution similarity of Histone H2A, Tubulin α-1A, and GFAP may suggest the correlation of the first two proteins in glioblastoma development or progression.

IMS-ROI analysis was performed to test the difference between tumour and normal ROI intensity for each batch of imaging data^12^. When analysis was performed with separate ROIs assigned to the tumour region and the normal region of the brain tissue, molecules specific to the tumour region were detected. The parametric Student’s *t*-test achieved a match with a significance threshold of *P* < 0.01, and the *d*-value was adopted as before. IMS-ROI analysis was also used to compare disease and normal region protein intensities to determine potential biomarkers in tumour tissue. As shown in Tables 2 and S1, and Fig. 7 (sample 1), the intensities of histones H2A, H4, and H2B identified by ROI analysis have a higher average value, and a higher standard deviation of the signal intensity in the tumour region than in the normal region. The intensities of Tubilin α1-A and GFAP have the same standard deviation in the normal region as in the tumour region, and the mean value is higher in the normal region. In contrast, the three histones, H2A, H4, and H2B, having large standard deviations in the tumor region may represent heterogeneity of the metastatic tumour. This is probably a characteristic of small cell carcinoma tumour tissue. It is highly likely that other proteins show the same level of strength because they are uniformly expressed in the normal tissue, and they are not related to tumours. However, in glioblastoma in sample 3, H2A, H4, Tubulin β-2A, Tubulin α-1A, and GFAP show more stable higher intensities than in the normal region, suggesting a difference between metastatic lung tumors and glioblastoma.

Our study is limited to the small number of patients, and the data do not strongly determine the extent of brain tumour. Nevertheless, IMS shows a correlation between GFAP and the brain tumour markers H2A and Tubulin α-1A, suggesting tumour development. In the future, more brain tumour markers will be identified by IMS combined with IMS-HCA and IMS-ROI. In fact, reducing image margins is important for minimizing mismatches in the amount of peptide in the sample. This is worth considering in future researches. As the development of this platform progresses, we will report on this elsewhere.

## Methods

### Patients

This study and all its protocols were approved by the Medical Ethics Committee of the Graduate School and Faculty of Medicine, Kyoto University, Japan. We obtained informed consent from all study participants, who donated their tumours in return for receiving its surgical excision. All experiments and image data analyses were carried out according to the relevant guidelines and regulations, including Ethical Guidelines for clinical studies by the Ministry of Health, Labour, and Welfare, as well as the Ministry of Education, Culture, Sports, Science, and Technology. The pathological diagnosis of all excised tumours was either glioblastoma or SCLC.

### Tissue preparation

Brain tissue was prepared with 10% (v/v) formaldehyde in phosphate buffer (pH 7.2) immediately after excision. After fixation, tissues were embedded in paraffin. These paraffin-embedded blocks were sliced into 4 μm sections for microscopic observation with haematoxylin and eosin.

FFPE tissues were deposited on indium tin oxide-coated (ITO) glass slides (Sigma–Aldrich, St. Louis, MO) and were treated in 800 μL of the pre-treatment buffer (0.1 M NH_4_HCO_3_ and 30% (v/v) CH_3_CN) in an incubation chamber for the brain tissues^2^. Incubation in pre-treatment buffer, heating, and digestion with trypsin solution including 2.5 mM NH_4_HCO_3_ and 10% (v/v) CH_3_CN were added to the chambers as described previously in detail^2,12–14^. By these incubation and steaming procedures (SSP), we succeeded in increasing the ionization efficiency of the protein on the FFPE specimen to approximately 4–5 times of the conventional method, and reducing a nonspecific signal noise^2^. Without the SSP method, we could not have gained the sufficient intensity form of the ionized peptides in the current study.

### Matrix deposition

Four sample slides were placed in a slot on a MALDI target plate and attached with conductive tape. The prepared matrix solution was 2,5-dihydroxybenzoic acid (50 mg/mL) in 50% methanol and 0.05% trifluoroacetic acid. The matrix solution was added to the sections using a CHIP-1000 chemical inkjet printer (Shimadzu, Kyoto, Japan) with a droplet size of 5-nL by micro-spotting in 25 cycles of 200 pL per spot at a spatial distance of 250 μm. After spotting, the target plate was dried in a desiccator at 20 °C.

### Tandem MS, statistical analysis and MS imaging (IMS)

We collected MS/MS data, using a MALDI-QIT-TOF MS (AXIMA Resonance and MALDI-7090; Shimadzu) equipped with a 337 nm pulsed nitrogen laser run at a frequency of 10 Hz for gaining data of sample 1 for the identification of the protein in IMS. Thereafter, we exported the spectra to the Mascot search engine (Matrix Science, Boston, MA), using the following parameters: taxonomy = Homo sapiens; MS/MS tolerance = 0.3 Da; enzyme = trypsin; missed cleavage = 1: database = SwissProt; and MS tolerance = 0.2 Da. We identified peptides and proteins, using the Paragon algorithm provided with ProteinPilot 4.5 Beta (AB SCIEX, Danaher, Washington, D.C.) combined with the UniProt-Swiss-Prot database (version 2010–6, Homo sapiens). We assigned matches to a significance threshold of P < 0.0513. False discovery rate (FDR) analysis was performed after using the Proteomic System Performance Evaluation Pipeline (PSPEP) software (Danaher). We quantified peptides, using Protein Quantitation 1.0 MicroApp (PQMA). Moreover, we analyzed liquid chromatography-tandem mass spectrometry (LC-MS/MS) datasets for all samples, using ProteinPilot 4.5 beta. The data file was imported to the Peak View 1.1.1 platform, using PQMA. Protein abundance was obtained, using Marker View 1.2.1. We selected peptides with a confidence > 0.95 for export. The *m/z* values corresponding to the proteins identified in tandem MS of sample 1 were assigned with two decimal places equal to the spectra of samples 2–4. The spectra were recorded in positive ion mode in a *m/z* range of 700–3000. Calibration was performed with a mixed solution of angiotensin II, and adrenocorticotropic hormone fragment. The pixel sizes are: Sample 1: 1180 pixels, Sample 2: 861 pixels, Sample 3: 1125 pixels, and Sample 4: 1116 pixels.

### Protein extraction and LC/MS

The proteins identified in IMS were actually detected in the data of three brain tumor samples by LC/MS, and included in the higher coverage (%) (>5.0) group (Supplementary Table S2–4. The LC/MS measurement method at that time is shown below this section^32^. Samples were homogenized and suspended in 20 μL of 0.1 M NH_4_HCO_3_, containing 30% (v/v) CH_3_CN. After centrifuge, incubation, and cooling, samples were incubated in trypsin solution at 37 °C overnight. We added 10 mM DTT, and heated the digests at 95 °C for 5 min. After drying, we re-suspended them in 0.1% TFA containing 2% CH_3_CN. The samples were separated, using a nano-flow reverse-phase LC (NanoLC-Ultra System; Eksigent, Dublin, CA). We injected an aliquot of 5 μL of each sample into a trap column, and washed it for 10 min, using 0.1% formic acid. Peptides were eluted for further analyses, using Triple TOF 5600 system (AB SCIEX, Framingham, MA) with a nano-electrospray ionization source (NanoSpray; AB SCIEX, Framingham, MA). We performed MS/MS scans, using a collision energy of 35 kV with unit-resolution.

### IMS-ROI analysis

Tumour ROI on the pathological image was diagnosed by two pathologists and manually mapped on the *m/z* image. Intensity values for all pixels in the ROI (tumour or metastatic tumour focus region) were represented by *m/z* images of each peptide. In IMS-ROI analysis, *P* value was evaluated by Student *t*-test and Cohen’s *d* value by $d=({s}_{i}-{s}_{h}){((({n}_{i}-1){{\sigma }_{i}}^{2}+(nh-1){{\sigma }_{h}}^{2})/({n}_{i}+{n}_{h}-2))}^{-1/2}$ where *s*_*i*_ and *s*_*h*_ represent the average signal strength of the ROI and normal region pixels, respectively. *n*_*i*_ and *n*_*h*_ are the ROI and normal pixel numbers, respectively. *σ*_*i*_ and *σ*_*h*_ represent the standard deviation of pixel ROI and normal region intensity, respectively^33,34^. Cohen’s criteria are as follows: *d* < 0.2, not important. 0.2 < *d* < 0.5, small; 0.5 < *d* < 0.8, medium; *d* > 0.8, significant. We determined that there was an appreciable difference between the normal region and the tumour region when 0.5 < *d*.

### Hierarchical cluster analysis (HCA)

MS Solution v1.20 (Shimadzu) was used for both IMS-HCA with the Ward method and the group average method. When HCA was performed, the Euclidean distance between the data matrix of each image was measured. The images that were close together were combined into one large cluster, and then the distance between each newly grouped individual cluster was calculated. By repeating this process, clustering was performed hierarchically on the dendrogram. The vertical axis represents distance, and the *m/z* value was represented on the horizontal axis.

## Supplementary information

Supplementary Information.

## Data Availability

The datasets generated and/or analysed during the current study are available from the corresponding author upon reasonable request.
