# Supplementary material for: Hierarchical Cluster and Region of Interest Analyses Based on Mass Spectrometry Imaging of Human Brain Tumours
Source: Sci Rep. 2020 Apr 1;10:5757. doi: 10.1038/s41598-020-62176-8 (PMC7113320; doi:10.1038/s41598-020-62176-8)
Supplement: Supplementary file 1 — Supplementary Information. [file 41598_2020_62176_MOESM1_ESM.pdf]

Supplementary materials

Hierarchical Cluster and Region of Interest Analyses Based on Mass Spectrometry Imaging of Human Brain Tumours

Takuya Hiratsuka, Yoshiki Arakawa, Yuka Yajima, Yu Kakimoto, Keisuke Shima, Yuzo Yamazaki, Masahiro Ikegami, Takushi Yamamoto, Hideshi Fujiwake, Koichi Fujimoto, Norishige Yamada, Tatsuaki Tsuruyama

Table S1: Comparison of the signal intensities of the peptides in tumour region
SD: standard deviation

**Sample 1 *m/z***	**average intensity**	**SD**
Histone H2A (*m/z* 945)	3015.8	1777.8
Histone H4 (*m/z* 1181)	917.3	500
Histone H2B (*m/z* 1744)	589.9	287.1
Tubulin β-2A (*m/z* 1621)	693.4	355.5
Tubulin α-1A (*m/z* 1703)	452.6	188.1
GFAP (*m/z* 1209)	402.1	150.5
**Sample 2 *m/z***	**average intensity**	**SD**
Histone H2A (*m/z* 945)	425.7	129.3
Histone H4 (*m/z* 1182)	610.6	217.9
GFAP (*m/z* 1210)	906.4	367.1
GFAP (*m/z* 1216)	873.8	356.4
**Sample 3 *m/z***	**average intensity**	**SD**
Histone H2A (*m/z* 945)	316.6	134.3
Histone H4 (*m/z* 1181)	258.6	106
Tubulin β-2A (*m/z* 1621)	368.9	202.2
Tubulin α-1A (*m/z* 1703)	334.7	177.8
GFAP(*m/z* 1209)	337.7	201.5
GFAP (*m/z* 1216)	267.5	149.4
GFAP (*m/z* 1264)	324.8	236.7
HBA (*m/z* 1530)	220.8	315

**Sample 4 *m/z***	**average intensity**	**SD**
Histone H2A (*m/z* 946)	740.5	357.5
Histone H4 (*m/z* 1182)	455.9	213.5
GFAP *m/z* (*m/z* 1209)	783.1	460.7
GFAP (*m/z* 1216)	492.7	227.4
GFAP (*m/z* 1264)	494.6	268
HBA (*m/z* 1530)	666	721.7

Supplementary figures

Supplementary fig.1

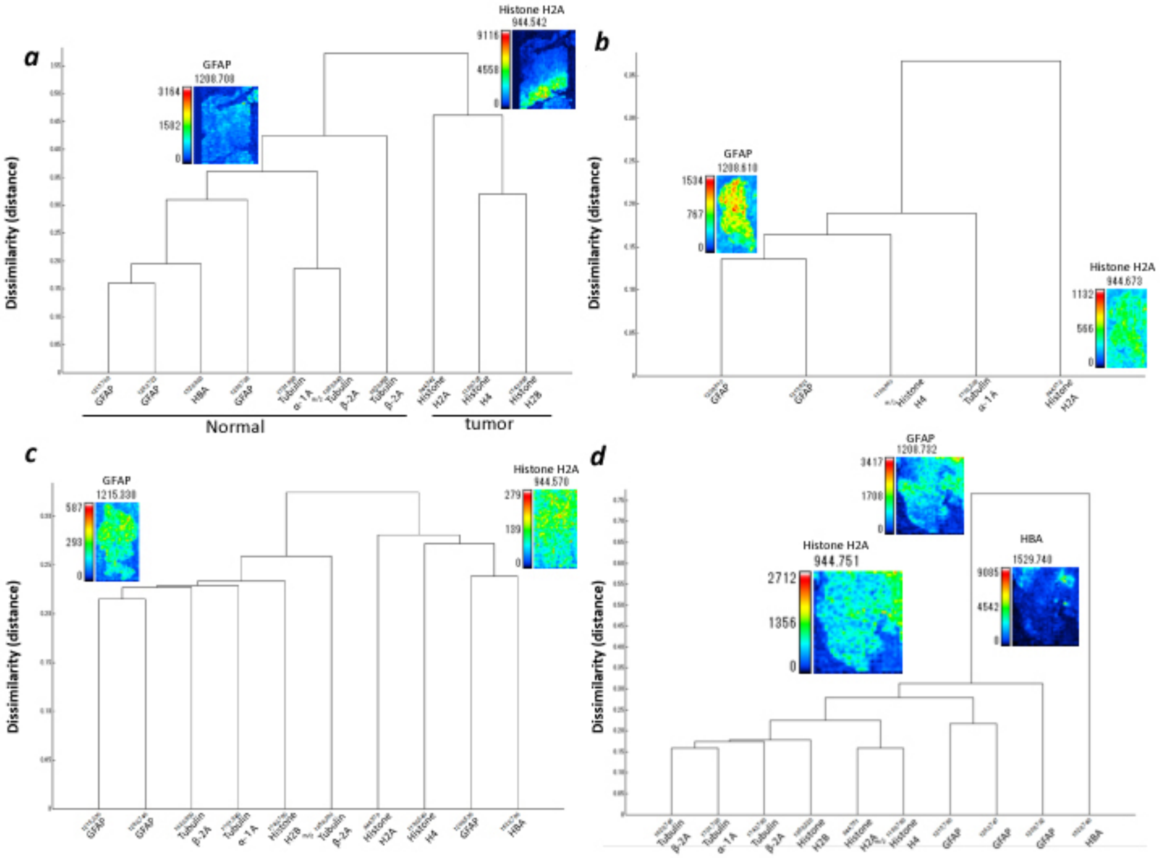

Supplementary fig.1

Cluster analysis of the two-dimensional distribution of the selected peptides. The data from two-dimensional distributions were used to calculate a cluster dendrogram. The vertical axis represents dissimilarity, and horizontal axis represent the *m/z* values. HCA analysis, with group average method **(*a*)** sample 1, **(*b*)** sample 2, **(*c*)** sample 3, **(*d*)** sample 4.

Supplementary fig.2

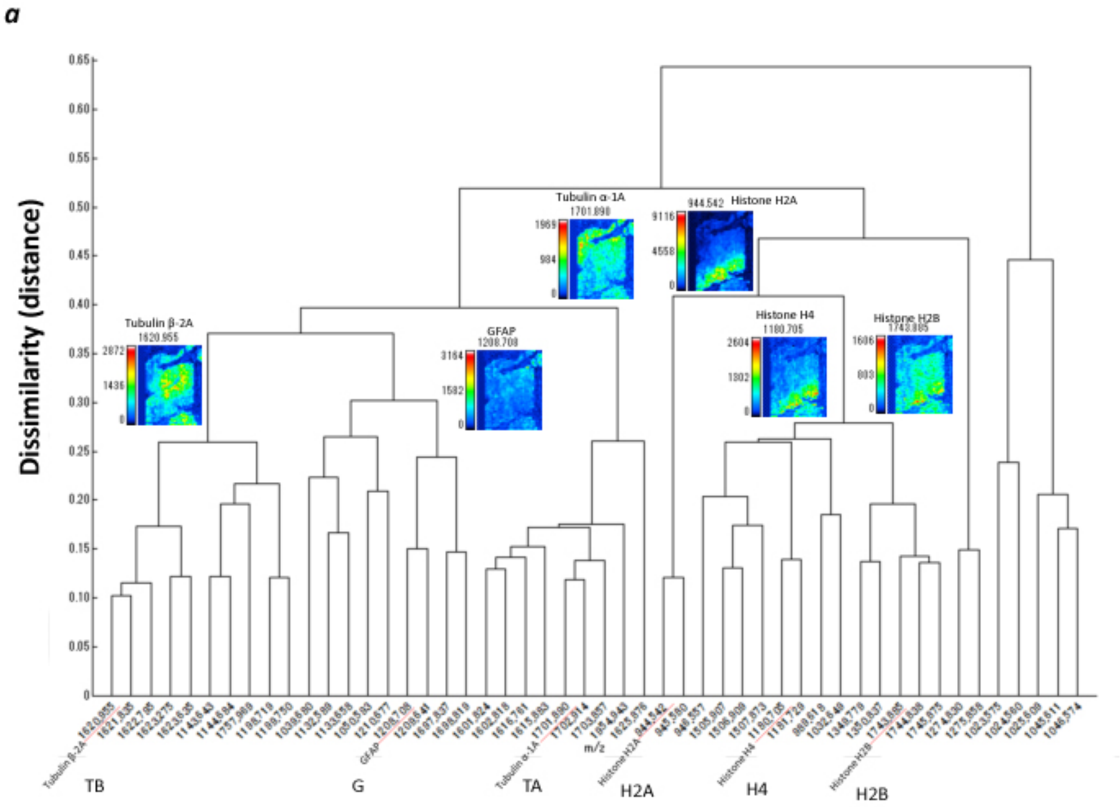

b

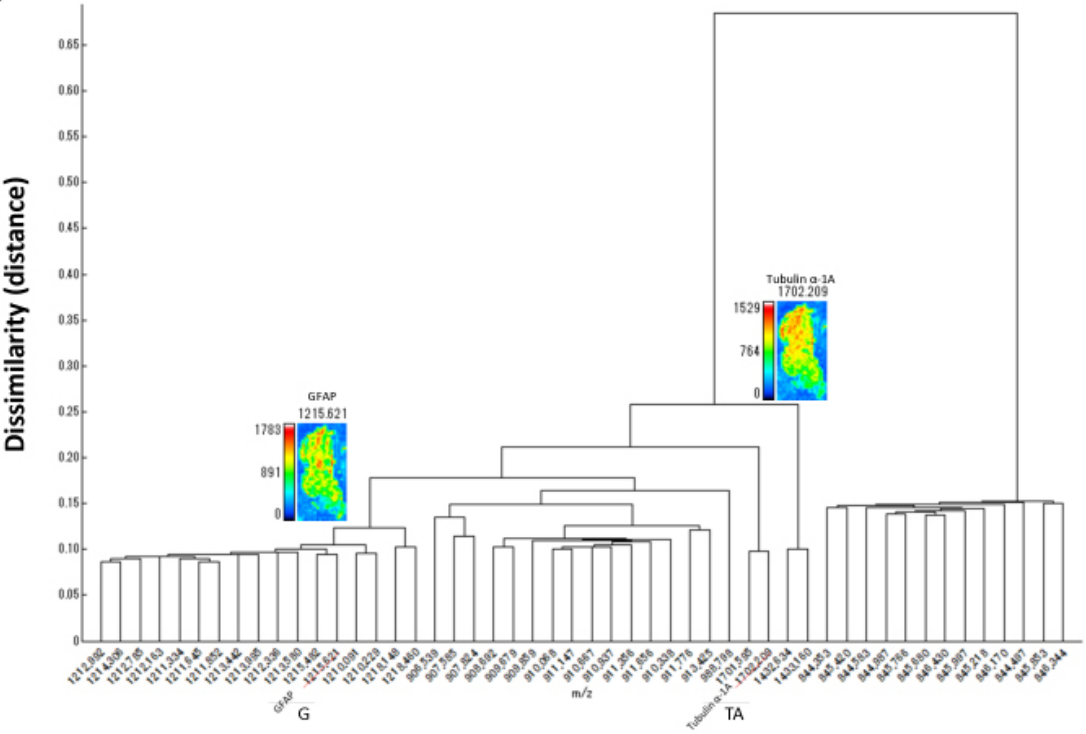
Dissimilarity (distance)
0.65
0.60
0.55
0.50
0.45
0.40
0.35
0.30
0.25
0.20
0.15
0.10
0.05
0
GFAP
1215.621
1783
891
0
Tubulin α-1A
1702.209
1529
764
0
m/z
GFAP
G
Tubulin α-1A
TA

c

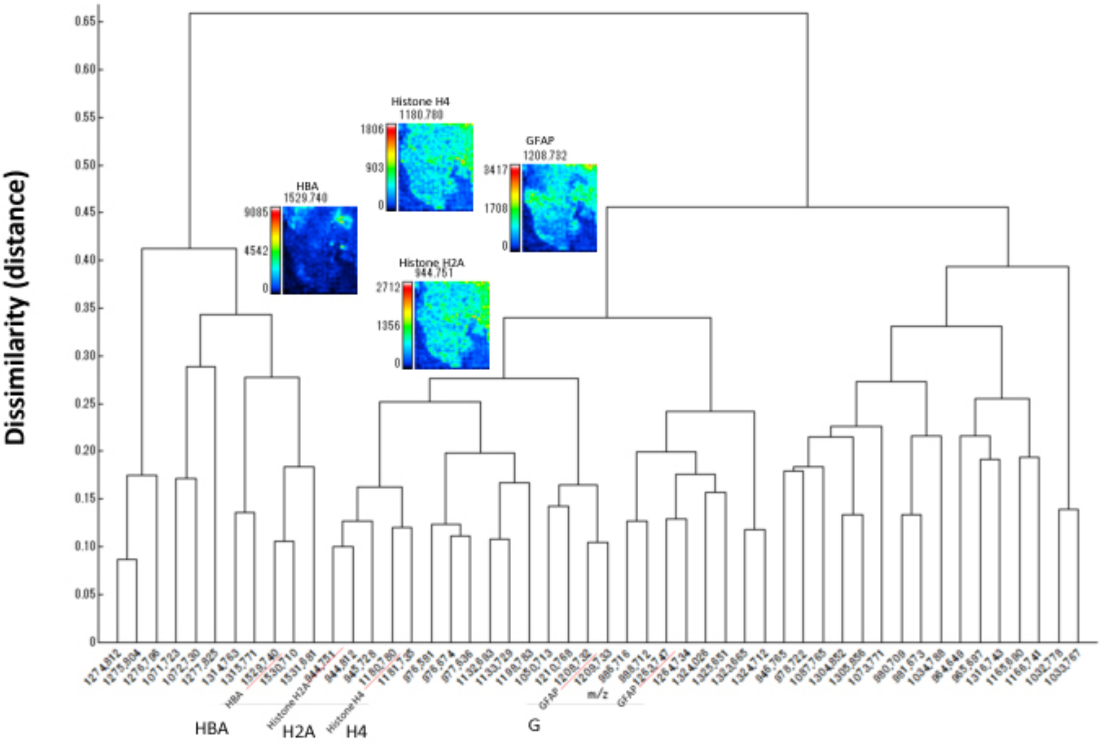
Dissimilarity (distance)
0.65
0.60
0.55
0.50
0.45
0.40
0.35
0.30
0.25
0.20
0.15
0.10
0.05
0
HBA
1529.740
9085
4542
0
Histone H4
1180.700
1806
903
0
GFAP
1208.782
3417
1708
0
Histone H2A
944.751
2712
1356
0
m/z
HBA
Histone H2A
Histone H4
GFAP
HBA
H2A
H4
G

Supplementary fig.2

Cluster analysis of the two-dimensional distribution of the whole spectrum.

The data from two-dimensional distributions were used to calculate a cluster dendrogram. The vertical axis represents dissimilarity, and horizontal axis represent the *m/z* values. HCA analysis with group average method: **(*a*)** sample 1, **(*b*)** sample 2, **(*c*)** sample 4. HB, HBA; TB, Tubulin β-2A, G, GAFP; TA, Tubulin α-1A; H2A, Histone H2A; H4, Histone H4. Histone H2A.
